# Ethics and Occupational Health in the Contemporary World of Work

**DOI:** 10.3390/ijerph15081713

**Published:** 2018-08-10

**Authors:** Sergio Iavicoli, Antonio Valenti, Diana Gagliardi, Jorma Rantanen

**Affiliations:** 1 Department of Occupational and Environmental Medicine, Epidemiology and Hygiene, Italian Workers’ Compensation Authority (INAIL), Via Fontana Candida 1, Monte Porzio Catone, 00078 Rome, Italy; s.iavicoli@inail.it (S.I.); d.gagliardi@inail.it (D.G.); 2 Department of Public Health/Occupational Health, University of Helsinki, P.O. Box 20 (Tukholmankatu 8 B), FI-00014 Helsinki, Finland; jorma.h.rantanen@gmail.com

**Keywords:** changing world of work, code of ethics, ethical dilemmas, ethics, occupational health, occupational health professionals

## Abstract

The last two decades have seen increasing attention to professional ethics in the field of occupational health in industrialized and developing countries, partly reflecting the changing world of work, demographic shifts and new technologies. These changes have led to the revisiting of traditional ethical principles and the emergence of ethical issues related to occupational health. This article looks at the problems raised by these ethical concerns and proposes some solutions. We revised the existing literature on the ethical conflict in occupational health in order to identifying drivers and barriers for correct professional ethics. The ethical choices are not only based on balanced risk and benefit assessment for various stakeholders, but there are a number of deontological aspects as well that go beyond the mere benefit domains. There is still no systematic approach for analysing the true extent of these issues and their solutions.

## 1. Introduction

Over the past decades many new issues have come to the fore, reflecting changes in the world of work, fragmentation, economic difficulties, demographic shifts, new technologies and, more generally, the impact of globalization. The workforce itself is also diversifying, with an increasing participation of women, migrants and older workers [1,2]. A growing body of literature on the impact of new forms of work organization (e.g., changes in management methods, use of contractors and temporary staff, changes in working hours and increased ability to work away from a fixed workplace or from home, etc.) highlighted negative effects on workers’ health and safety. The organization of work itself can influence the level of psychological stress that workers experience and can increase health problems (e.g., musculoskeletal disorders, cardiovascular disease and metabolic syndrome and diabetes) related to exposure to occupational hazards, which can lead to injuries or illnesses [3,4].

These call for radical changes in occupational health, considered an important element in the social dimension of working life, in line with a broader concept of global and integrated promotion of well-being at work, giving rise to many new challenges for workers and their representatives, employers, managers, health service providers, government authorities, professional associations and social partners alike.

A new paradigm for occupational health has emerged, extending the classical focus on “health risk management” to include also the medical aspects of sickness absence and rehabilitation, the support and management of chronic non-communicable diseases, and workplace health promotion. In the last decades, the overlap between the two domains “prevention” (of hazards) and “promotion” (of health) has become greater. At present, “occupational health” is meant to include not only health protection, but also health promotion in the workplace.

A very central role in this scenario is played by the occupational health professionals (OHPs) due to the increasingly complex and sometimes competing responsibilities of OHPs towards workers, employers, the public, public health and labor authorities and other bodies such as social security and judicial authorities. In the coming years, the occupational physicians should be able to act as a care provider and an expert, a decision maker, a communicator and a counsellor, a leader and an advisor, a manager. New challenges and new ways of working will necessitate a review of the competence and capacity of the OHPs, with implications for future workers’ health and safety [5,6,7].

The term OHPs is used here to include all those who, in their professional capacity, carry out occupational health and safety tasks, provide occupational health services or are involved in an occupational health practice. OHPs therefore include occupational health physicians and nurses, factory inspectors, occupational hygienists and occupational psychologists, ergonomists, specialists in rehabilitation therapy, in accident prevention and in the improvement of the working environment, as well as those developing occupational health and safety research [8].

This scenario is setting new contexts for OHPs in their tasks (e.g., health surveillance, workplace monitoring, interventions to prevent of health hazards, assessment of employees’ ability to work, pre-employment health examinations, risk assessments, rehabilitation of workers with health disorders, etc.) and their professional conduct. Health professional has simultaneous obligations, either explicit or implicit, to a third party, the consequences of which may lead to adverse impacts on a patient, client, or client community.

In particular, in recent years, increased regulatory complexity, emerging issues related to diagnostic tests that may violate the ethical sphere or to technological innovation, increased the complexity of the decision-making process, emphasizing the role of health professionals in balancing between the individual good (i.e., the health and working capacity of individual employees) the good of the enterprise (i.e., maximize the production) and the common good (i.e., the health and safety of the community at large).

OHPs are faced with many ethical dilemmas concerning aspects such as respect for confidentiality and consent to data processing, the choice of tests for health surveillance, workers’ right to know and the autonomy of workers’ decisions, the transparency in relationships between colleagues and the maintenance of defined professional quality standards [9].

However, the ethical choice in the era of globalization has become even more complex (due to a growing number of potential stakeholders) and pluralistic, accounting for history, culture, religion, value systems and social practices. Examples include the conflict between the right of workers to life, health and safety and the right of businesses to maximize production, or between the right of workers to be informed of risks and the right of businesses to industrial secrecy, and the rights and duties of OHPs [10].

Yet another radical shift is the development of a multidisciplinary approach in occupational health (i.e., technical, medical, social and legal), which implies the involvement of other specialists who belong to various professions in occupational health services [8,11].

This article reviews the development of the concept of ethics in occupational health in the contemporary world of work, analysing emerging ethical concerns and offering some proposals for solutions.

## 2. Materials and Methods

We searched for potentially eligible studies in a grey and peer-reviewed literature databases, internet search engines, organizational websites and major electronic academic databases.

The search strategy was built into PubMed syntax using the following key words (both as MeSH terms or quoted sentences and loose words in the title and abstract field): biomedical ethics; bioethical issues; code of ethics; human right; occupational health; occupational health physician; occupational health nursing; workplace; risk assessment; risk management; globalization; challenges; conflict; dilemma; concern; choice; solution. Provided the syntax adaptation, PubMed and Scopus databases were searched.

Additionally, scientific journals, books and conference proceedings relevant to the topic were analysed with a view to identifying the major achievements in the field, the main areas of debate and the outstanding research questions. Two basic inclusion criteria were used. The first set includes criteria pertaining to publication characteristics, such as full-text article publication (not just an abstract), peer-reviewed publication, English-language publication, and exclusion of duplicate publications. The second set includes criteria pertaining to study design, study conduct and reporting, and study relevance to the key question(s) concerning the relationship between ethics, occupational health and changing world of work. 

The main aim of this study is to identify drivers and barriers to appropriate professional ethics, with a special focus on the ethical conflicts in the field of occupational health.

To achieve this objective, we adopted a stepwise approach. Initially, we focused on the impact of the changing world of work on new contexts for OHPs in their tasks (e.g., health surveillance, workplace monitoring, and interventions for the prevention of health hazards). In particular, we paid attention to the emerging ethical issues related to technological innovation and the globalization of business (business ethics).

Afterwards, we reviewed in detail the ethical dilemma in occupational health. Starting from the first bioethics studies in the 70s, we analysed the ethical dilemma from the individual, professional and institutional point of view; for each one, we considered aspects such as person/body involved, environment of operation, philosophical basis, field of application, value content, learning arena and guidance. Finally, we carried out a comprehensive review of the literature on ethical decision-making models in order to identify strengths and weaknesses that affect the development of ethical choices.

## 3. Results

### 3.1. Ethics and Occupational Health: Historic Roots

About 2500 years ago, a Greek philosopher and doctor, Hippocrates, crystallized the key principles of professional ethics in his famous Hippocratic Oath, which has survived through centuries and still constitutes the core of medical ethics [12].

Traditionally, the historical roots of the relationship between ethics and occupational health date back to the classical world, in particular to Plato (427-347 AD) who provides one of the first ethical considerations on the doctor-patient relationship (DPR) in a famous passage of his last work “Laws” [13].

In the 11th century, Western medical ethics inherited the moral values of Catholicism and the focus shifted to the duties and principles a good doctor must adopt in his professional conduct. As an example, the Italian physician Gabriele Zerbi (1445–1505) in his *De Cautelis Medicorum* (1495)—the first systematic account of medical ethics—stated that the physician is like a priest to whom God has revealed the divine powers of healing and to whom men reveals their souls for the cure of their bodies [14].

The Lutheran reform in the 16th century again changed the concept of work. Working hard, in a disciplined and productive way, became a call from God. But the call did not involve the idea of enjoying the fruits of work; a frugal lifestyle was adopted as an ethical norm, and in fact was expected to lead to the accumulation of capital, to economic growth and to the generation of security and wealth. Occupational health and safety (OSH) were not aims on their own, but the results of the economic rationale of avoiding loss of productive working time—a trend that is clearly dominant, for example, in the current EU OSH policies. The concept of wellbeing at work came very late on the agenda—not much earlier than at the beginning of the 21st century [15,16].

Bernardino Ramazzini made a large personal contribution to the field of occupational medicine by collecting his observations in *De Morbis Artificum Diatriba* (1700). In its Preface, he explained the ethical and social reasons why physicians and society are expected to take care of workers’ health. Ramazzini’s moral attitude towards workers’ health is based on the so-called ethics of the two virtues: compassion and gratitude. As to compassion, Ramazzini believed that “it is necessary to admit that the workers in certain arts and crafts sometimes derive from them grave injuries, even the death”. As to gratitude, Ramazzini said that “I publish an imperfect work to the wretched conditions of the workers from whose manual toil, which is often extremely laborious and degrading, albeit necessary, so many benefits to mankind accrue”. Also, he demonstrates a sense of justice (“...in our own time also laws have been passed in well-ordered cities to secure good conditions for the workers; so it is only right that the art of medicine should contribute its portion for the benefit and relief of those for whom the law has shown such foresight”) [17,18].

As he would have wished, Bernardino Ramazzini’s thinking and practice were reflected far beyond the occupational and public health field and not only in the medical professions, but the whole social and economic spheres. As an example, Adam Smith cites Ramazzini in his work “An Inquiry into the Nature and Causes of the Wealth of Nations” (1777). He states that “almost every class of artificers is subject to some peculiar infirmity (..); if masters would always listen to the dictates of reason and humanity, they have frequently occasion rather to moderate than to animate the application of many of their workmen”. He also rejected the idea that “men work better when they are ill fed than when they are well fed, when they are disheartened than when they are in good spirits, when wages are low than when they are high” [19].

In the following century, Karl Marx used Ramazzini’s work to describe workers’ conditions. In his most important work, Capital (1867), Marx argued that “the division of labor in manufacture affects the very root of individual life and it is the first to afford the materials and impetus for industrial pathology” [17].

With regard to socially weaker groups, some years later, Pope Leo XIII in his Encyclical *Rerum Novarum* (1891) argued that “a work which is quite suitable for a strong man cannot rightly be required from a woman or a child (..) women, again, are not suited for certain occupations; a woman is by nature fitted for house-work and it is that which is best adapted at once to preserve her modesty and promote the good bringing up of children and the well-being of the family” [20].

Over the centuries, his belief has had a substantial economic, social and philosophical impact on the relations between health and work and this is reflected by some 18th and 19th century figures [21].

In the 20th century, bioethics too laid particular emphasis on the physician-patient relationship, highlighting its main characteristics and identifying critical issues by stating principles of mutual responsibility, solidarity, fairness, tolerance as well as by calling for a just and sound treatment of each and any individual, including provision of honorable working conditions [22].

In times of “laissez faire” and deregulatory policies, one needs to be reminded that public institutions should consider themselves servants for those “sovereign consumers” who are unable to pay for them. In developed societies, and even more in developing ones, the pure market society is the problem rather than the solution. Political institutions in a democratic society are justified precisely by the fact that they are meant to protect the members of that society from humiliation generated by the market society. This includes safeguards against poverty, homelessness, exploitation of workers, degrading work conditions, and the unavailability of education and health. Such principles can only be achieved where there are strong societal, business and institutional ethics, fostering and enabling individual ethical choices [23,24].

Thus, the traditional DPR reaches out to other groups of people, and into several levels of the societal systems, as far as policy-making, government and other public agencies, corporations and institutions, non-governmental organizations and professional associations beyond medical communities. Many non-medical professional organizations have already responded by drawing up ethical codes of conduct. It is worth noting that their guides include the Hippocratic-Ramazzinian principles. Then, about 50 years ago, the concept of institutional ethics under the heading of Corporate Social Responsibility (CSR) was introduced, as guidance for the activities and decision-making of institutions, enterprises and other organizations [25].

### 3.2. The Dilemma of Ethical Choice

The presence of innumerable variables to be taken into consideration in the ethical choice produces what is called conflict or ethical dilemma, which can be defined as a decision making problem between two possible moral imperatives, neither of which is unambiguously acceptable or preferable [26,27]. Dilemmas may arise out of various sources of behavior or attitude. For instance, dilemmas may arise out of failure of personal character, conflict between personal and professional values, or even between them and those of a particular patient or society.

The failure to identify these dilemmas may have several consequences to the point of infringing a worker’s fundamental human rights [28], that are recognised in various international and regional human-rights treaties (e.g., International Covenant on Economic, Social and Cultural Rights; International Convention on the Elimination of All Forms of Racial Discrimination; Convention on the Rights of Persons with Disabilities), as well as in national constitutions, domestic laws, policies, and programmes [29].

In the 1970s the ethical conflict in the practice of occupational medicine has received attention from a bioethics perspective seeking to advance research and scientific development to defend and improve the living standards of human beings, and especially bound to identify problems of ethical dimension arising in healthcare [30].

In 1979, Beauchamp and Childress discussed the basic set of four Hippocratic values or ethical principles that can provide guidance in analysing and resolving ethical issues [31]:

- Autonomy: based on the principle of respect for persons, which holds that individuals have the right to make their own choices and develop their own life-plan defined as a person’s ability to make his or her own decisions. Autonomy occupies a substantial space in Western, and particularly US-oriented cultures, while countries with more of the Confucian traditions of East Asia tend to have more community-oriented and duty-based traditions, leaving more room for harmony and consensus and less for individual autonomy. In Islamic medical ethics, greater emphasis is placed on beneficence than on autonomy, especially at the time of death [9]. In a health care setting, the principle of autonomy translates into the principle of informed consent defined as “autonomous authorization of a medical intervention by individual patients”, which began to play a central role in clinical and research ethics in the twentieth century when problems related to autonomy of subjects became more persistent. Although still controversial, the current relevance of informed consent is evidenced by the fact that almost all the agreements and declarations that set out ethical and legal standards in the biomedical sector, consider it as an important component of medical profession and scientific research as well as of a successful DPR [32,33,34].
- Beneficence (*primum non nocere*): is the classical cornerstone of bioethics and medical ethics. The ordinary meaning of this principle is that health care providers have a duty to benefit the patient, while also taking steps to prevent or remove harm to the patient.
- Non-maleficence*:* implies avoiding the causation of harm—the healthcare professional must not harm the patient. However, as many medical treatments involve some harm, even if minimal, it should never be disproportionate to the benefits.
- Justice: defined as a form of fairness or, as Aristotele said, “giving to each that which is his due”. According to this approach, costs and benefits should be equitably distributed. It is based on two opposing principles—likeness (equity) and difference. The principle of equity implies an obligation to ensure that all persons have a *prima facie* right to the greatest possible freedom of action, compatibly with all other persons having the same right regardless of ability to pay, age, sex, nationality, religious faith, ethnicity, etc. The principle of difference requires that dependent and vulnerable persons have a right to claim and obtain satisfaction of needs if their own efforts fail to achieve this [9,25].

Other values too can be used as criteria or tools in ethical analyses. These fall under the headings of human rights, solidarity, cost-benefit or cost-efficiency, or harmony [31].

Since the 1990s several criticisms have arisen regarding bioethical principles. Clouser and Gert stated that principlism lacks systematic unity, thereby creating a practical and a theoretical problem [35,36]. Garrafa and Porto questioned the lack of a practical ethical intervention in principlism, especially when it comes to solving problems arising from economic and social inequality [37]. Petrini, on the other hand, stated that the main criticism of bioethical principles derives from the difficulty of “balancing” between values [38].

In the following decades, some studies were carried out to investigate the extent and nature of the ethical conflict in the practice of occupational medicine. In the United States, for example, a study based on survey of the American Occupational Medical Association’s members was conducted. Its results indicate a strong reliance on traditional medical role models in responding to ethical conflict; 69% of respondents thought ethical dilemmas arose sometimes, involving, in particular, responsibilities to themselves and the patient (over 60% of responses), confidentiality, cost and acceptability of risk. Professional code of ethics and personal beliefs are considered the most useful in resolving conflicts [39].

In Finland, the results of a survey conducted among 200 occupational physicians and nurses revealed that nearly all (97%) reported situations involving ethical problems in their work, most often when the employer was reluctant to improve the working conditions, or when either the patient or the employer disagreed on assessed work ability. The use of methods lacking scientific evidence was listed as a cause for ethical concerns by 16% of the physicians and 7% of the nurses [40].

**Figure 1:**
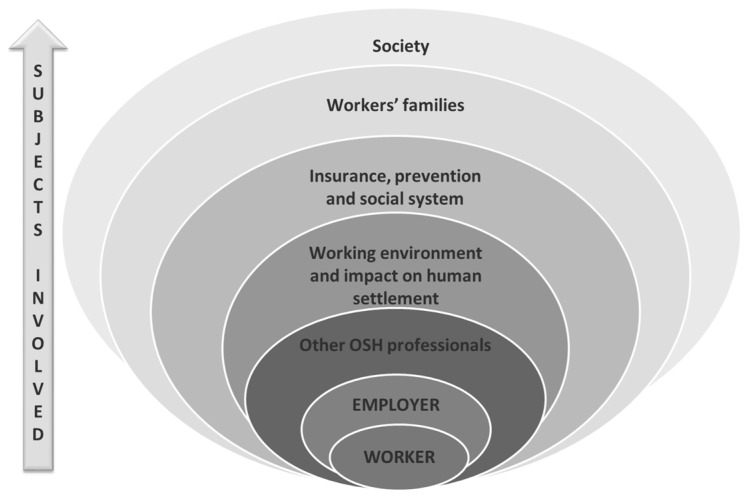
Subjects involved in decision-making by an occupational health physician**.** Source: modified from Brandt-Rauf, 1989 [39].

According to many studies, the ethical dilemmas for OHPs derive from the fact that, unlike DPR, OHPs have diverse roles, loyalties and responsibilities in the occupational health setting, the consequence of which may lead to adverse impacts on patient, employer, workers, government, companies, medical professionals and society at large (multiple loyalties) (Figure 1) [41,42,43].

In particular, an interesting study conducted by London shows that violations of the worker-patient’s human rights might arise from the incompatibility of simultaneous obligations; pressure on the professional from a third party and separation of the health professional’s clinical role from that of a social agent [28].

Confidentiality, objectivity, integrity, conflict of interest, communication about health hazards are some of the principles that physicians must take into account in their relationships with the individuals they serve, with employers and workers’ representatives, with colleagues in the health professions, and with the public. Some of these are well described in Ethics guidance for occupational health practice, an ethics guidance from the UK Faculty of Occupational Medicine (FOM) [44,45].

According to a shared opinion, without an ethical foundation, these principles are not sufficient to make an adequate judgment because they can be interpreted differently, leading to different results. An explanatory example might be the case of medical secrecy about HIV infection of a couple. If the doctor does not inform the partner about the infection, the autonomy is interpreted as respecting privacy, but if he decides to report the infection, autonomy is not only the patient’s but also the doctor’s, who cannot submit to all the patient’s demands. In either choice, the other ethical principles have different interpretations; it is therefore advantageous to support the traditional principles with modern formulations [38].

Also, the ethical dilemmas confronting occupational medicine is becoming more complex, because the development of science, market rules, civil and deontological norms of health professions do not always provide up-to-date answers to the ethical questions arising in modern work. Modern work life, however, has introduced new dimensions. Legislation, collective agreements, social security provisions and the legal responsibilities of diverse parties—employers, trade unions, social security institutions and insurance—and national and international trade rules have brought in new factors to be considered in both ethical guidance and conduct [11,46,47].

Any act related to occupational health (health monitoring, supervision, policy making, etc.) involves ethical choices that are usually based on four variables: laws and regulations; professional norms, good practice guidelines, codes of ethics, silent knowledge; sets of values and culturally conditioned practices in communities/societies; personal values [48].

OHPs operate in the environments where the principles of medical ethics and the social and economic realities of working life are in potential contrast.

Besides individuals, also institutions and enterprises are examined and assessed for ethics in terms of their strategies, decision-making and practical activities. In business companies this goes beyond the enterprise itself and its staff, reaching out to the community (community ethics) and environment (environmental ethics) and even to ethical investing [49,50].

It is difficult to balance and satisfy the values, beliefs, opinions of individual actors involved in the ethical choice. As rightly stated by Westerholm “there is no law of nature or law of man to resolve such dilemmas. It is for the health professional to choose action or non-action on the basis of societal civic values, professional values and personal values in combination” [9,51].

Except for the elaboration of international guidelines and codes of ethics by international professional and scientific organizations [8,44], there are still a few models of ethical analysis of decision making in the professional practice of the occupational physician which have tried to identify strengths and weaknesses of ethical choices.

The majority of these models analyse the ethical choice in businesses. Among these, the most comprehensive synthesis model of ethical decision-making by an individual was proposed by Jones. It includes four critical stages: (a) recognizing the moral issue, (b) making moral judgment, (c) establishing moral intent, (d) engaging in moral behavior. The proposed model takes into account individual factors (e.g., experience and social condition), context (e.g., social consensus, temporal immediacy, proximity) and organizational (e.g., corporate) culture that can influence the four critical stages [52].

The model proposed by Franco, on the other hand, offers an ethical analysis of the decision-making in occupational health practice. According to Franco, occupational physician must consider two main elements: (a) the stakeholders involved in the decision-making process (e.g., company, worker, employer and other figures of prevention, social partners, community etc.); (b) the bioethical principles of beneficence, autonomy and justice. Franco states that the ethical decision must be made after carefully evaluating the ethical benefits and costs (intended in a broad sense) borne by the decision itself. The uncertainty of the occupational physicians in assessing their performance and inter-individual variability in taking on the same problem are the main barriers that hinder the entire decision-making process [53].

In the United Kingdom, an approach to understanding moral conflict in “dual obligation” doctors (including OHPs) has been worked out. It is based on three different models describing the interaction between doctor and worker, compared with the normal DPR, focusing on different levels of trust required, possible power imbalance and the fiduciary obligations [54].

In order to address the ethical dilemmas a growing number of professional associations and occupational groups adopted codes of ethics with the goal of guiding their members, protecting service users, and safeguarding the reputation of the profession [5,8,9,55,56].There is a great deal of literature dealing with the question to what extent ethical codes can achieve their desired objectives and the lengths to which professionals’ practice must go to ensure independence from management influence. The analysis of the main studies and research shows how the main problems associated with the ethical codes concern the gap between rules and articles of a general and abstract code and real life, the great number of ethical codes and guidelines being produced and the legal interpretation of ethical issues [57,58].

According to Westerholm, codes are commonly well intentioned, but most often not legally enforceable. They are also only rarely evaluated. They vary significantly in clarity, depth, emphasis, strength and relevance. The focus in most codes is on the individual health professional’s conduct, less on the conduct of organizations [9].

Anyway, the International Commission on Occupational Health (ICOH), the world’s leading non-governmental scientific society in the field of occupational health, with a membership of 2000 professionals from 93 countries, starting from the late 1980s discussed the adoption of an international code of ethics. ICOH Code of Ethics can be considered as the world standard in ethics for occupational health professionals in enterprises and in private and public sectors. It applies to many professional groups with tasks and responsibilities concerning safety, hygiene, health and the environment in relation to work, with a view to providing all professionals with guidelines in the field of occupational health and identifying a reference level for evaluating performance. According to ICOH Code, OHPs must protect workers’ health, promote respect for human dignity and encourage the adoption of the highest ethical principles in the application of occupational medicine strategies and programs. The integrity of professional conduct, impartiality, respect for professional secrecy and workers’ privacy are also part of these duties. Public and private institutions as well as organisations employing OHPs should adopt a programme of organisational ethics aligned with the ethical principles of ICOH Code (art. 19) [8,59].

The importance of the ICOH Code is reflected by the fact that national legislations (Argentina and Italy) [60], international organizations (ILO, CIOMS, WHO), and other codes of ethics, post-graduate courses or treatises contain references to the subject [2,26,61,62,63]. As far as the Italian national legal framework is concerned, pursuant to art. 39, c. 1 of Legislative Decree 81/08, “the activity of occupational physician is executed under the principles of occupational health and ICOH Code of Ethics” [64].

### 3.3. Ethical Concerns in a Changing World of Work

The changes in the nature and conditions of work, including globalization of organizations, demographic changes and introduction of new technologies, have given rise to many new challenges for workers and their representatives, employers, managers, health service providers, government authorities, professional associations and social partners, impacting at the same time the ways in which moral/ethical problems are manifested.

Lefkowitz advanced the thesis that those changes may affect the manifest nature of the ethical and moral problems to be expected, but the domain of moral action is unaffected; the capacity to identify these new dilemmas relies on personal attributes such as one’s moral values, sensitivity and ethical vigilance [11].

First, globalization of business and working life has modified the environment where the ethical principles are applied. As business became less fixed territorially, corporations increasingly engaged in overseas markets, suddenly finding themselves confronted with new and diverse, sometimes even contradicting, ethical demands; moral values, which were taken for granted in the home market, may get questioned as soon as corporations enter foreign markets. The intensification of the interconnection between persons and societies around the world has created the possibility that our actions could generate transnational or trans-border consequences [65].

From a managerial point of view, the ethical problems are related to the conflict between the organizational economic performance (evaluated by measuring turnover, costs and profits) and its social performance (evaluated by assessing the ethical responsibilities to the people outside or inside the organization). This implies an increasing prominence of health and work agendas, as improved work ability, reduced sickness absence and increased productivity are seen as central to economic success [66].

To manage these ethical problems, some scholars have suggested that the time has come for the world to develop a global ethic, i.e., a set of universally accepted principles that could provide the basis for regulating global interactions [65].

Therefore, international managers carry the heavy task of formulating organizational policies by combining the law, the local culture values, the ethical business principles and the organizational standards [67].

Further, increasing life expectancy and other demographic changes [68] affecting the labor market brought to the fore various ethical issues. First of all, studies have shown that older people are perceived as stereotypically rigid in thought and old fashioned in morality and values [69]; also OHPs working with older adults may face ethical dilemmas that they have not been trained or prepared to handle (e.g., age discrimination) [70]. A primary ethical issue is the preservation of the principles of autonomy, which may be affected by increasing physical disability and psychological conditions, which often lead to social isolation and depression [71].

However, as regards ethical concerns in a changing world of work, most studies have focused on ethical questions raised by modern technology within the fields of medicine and biotechnology. For example, particular ethical issues are emerging in the case of predictive genetic tests that require rules and behaviors that are completely different from those of standard ethical practice. The sensitivity of genetic tests compared to other biomedical examinations impose the need for greater control over the confidentiality of data. Considerable debate about ethical challenges of predictive genetic tests is whether public health professionals should inform family members of the results [72].

Other ethical aspects to be reassessed, in the case of predictive medicine, concern the decisional autonomy of the user, the balance between social responsibility and individual autonomy, respect for privacy, responsibility for the genetic health of future children, maximising social best interest/minimising serious social harm, etc. [73].

Also, the growing use of information and communication technologies (ICT) at the workplace have blurred the boundary between private and work life raising some ethical issues for employers, employees and their representatives, especially as regards the relationship between workers’ privacy and employers’ need to control and monitor the use of ICT [74], intellectual property [75], data quality [76], just to list a few.

Stahl has identified a set of ethical issues that are predicted to become relevant during the development of ICT [77].

Regarding the use of nanotechnology in the workplace, an interesting article by Schulte highlights how the lack of clarity about the hazards of nanomaterials and the risks they may pose to workers contributes to the emergence of ethical issues. According to Schulte, the main situations that have ethical implications include: (a) identification and communication of hazards and risks by scientists, authorities, and employers; (b) workers’ acceptance of risk; (c) selection and implementation of controls; (d) establishment of medical screening programs; and (e) investment in toxicological and control research. In the first place, it is difficult to understand what preventive measures must be taken and whether they comply with the bioethical principle of non-maleficence (*primum non nocere*), that is causing no harm. Furthermore, in the case of medical screenings, ethical questions arise regarding the voluntary nature of screening, access to results and the objective of such access. These problems are difficult to solve since the worker’s protection interests clashes with the needs of companies and society as a whole for a nanotechnology growth and development [46].

McGinn conducted a study among nanotechnology researchers, finding that most respondents agree that their ethical responsibilities are not limited to the laboratory environment of safety and integrity, but also extend to the society in which the research is carried out and is likely to be applied [78].

The past decade has seen a rapid growth of research in the area of ethics of robotics (roboethics), also and particularly as applied to healthcare [79]. “The target of roboethics,” as Veruggio and Operto explain, “is not the robot and its artificial ethics, but the human ethics of the robots’ designers, manufacturers, and users” [80].

The World Commission on the Ethics of Scientific Knowledge and Technology (COMEST) in an interesting report on robotics ethics [81], highlights that ethical issues in health and well-being sector are related to how these type of technologies are used in relation to care. Can robots provide care, what implications do they have for safety and security, and how do they influence our attitude towards ability and disability, and towards care for people who are ill, old, and vulnerable? In particular, given the increasing autonomy of robots, the arising question is who exactly should bear ethical and/or legal responsibility for robot behavior.

In recent years, three forecasting approaches to technology ethics have been formulated in order to anticipate ethical dilemmas at an early stage of technological development: ethical technology assessment (eTA) [82], techno-ethical scenarios approach [83] and ETICA approach [84]. Haris sets out a structured meta-methodology, named DIODE, for the ethical assessment of new and emerging technologies [85].

## 4. Discussion

Even though a growing body of literature highlighted the implications for ethics of OHPs reflecting the changing nature and conditions of work [2,3,4,5,6,7,9,10,11], demographic swifts [70,71] and technological innovation [54,72,73,74,75,76,77,78,79,80], we found that the studies about the procedures for addressing ethical issues in occupational health practice are still few [53]. Most studies concern the analysis of ethical choice in business and by an individual perspective [52] without considering, as rightly stated by Westerholm, the combination of social civic values, professional values and personal values [9]. Inspired by this consideration, we deal with the analysis of the main ethical challenges in occupational health through a multidisciplinary approach, taking into account medical, economic and social issues partly reflecting the changing world of work, demographic swift and the introduction of new technologies. We proposed an integrated approach to assessing the importance of all three types of ethics, personal (individual), professional and institutional, in resolving ethical conflicts. Another point of strength of our study is the identification of some drivers and barriers for correct professional ethics that can represent a starting point for the recognition of some proposals for ethical solutions. We identified drivers and barriers thanks to the ethical analysis of decision-making process in occupational health practice. This is an issue not always analysed in previous studies, which were limited to questioning individual issues such as informed consent [35,36,37], ethical issues in international business [22,47,66,67], corporate social responsibility [25], impartiality [41,42], confidentiality, integrity, conflict of interest, communication about health hazards [39], technological innovation, etc. [74,75,76,77,78,79,80,81,82,83,84,85].

However, the presence of numerous variables to consider (e.g., the conflict between the right of workers to life, health and safety and the right of businesses to maximize production; or between the right of workers to be informed about risks and the right of businesses to industrial secrecy; the rights and duties of OHP), as well as the growing number of potential stakeholders involved in ethical choice, prevented the identification of an ideal proposal able to solve ethical challenges in OHPs practice [9,51].

The present study, as already pointed out by the scientific literature, provides a critical evaluation of the four bioethical principles (autonomy, beneficence, non-maleficence, justice) considered not usable or meaningful guides, not coherently related in a “unified guide” and lacks a “single clear, coherent, and comprehensive decision procedure for arriving at answers” [86]. Without an ethical foundation, these principles are not sufficient to make an adequate judgment because they can be interpreted differently, leading to different results [38].

Furthermore, the present study emphasizes the importance of the OHPs in balancing the individual good (i.e., health and working capacity of individual employees), the enterprise’s good (i.e., maximize the production) and the common good (i.e., health and safety of the community) because of the increasing complex and sometimes competing responsibilities of OHPs towards the workers, the employers, the public, public health and labor authorities and other bodies. The balancing of the various interests, opinions, values and criteria in the complex multi-actor context remains as a truly difficult and unresolved area of dilemmas, as underlined in previous studies [11].

Another similarity with previous studies is the recognition of the limits of codes of ethics in solving ethical dilemmas due to problems of interpretation, problems of multiplicity of codes, problems of their legalization. Ethical codes vary significantly in clarity, depth, emphasis, strength and relevance. Also, the focus in most codes is on the individual health professional’s conduct, less on the conduct of organizations [26,51,57,58].

Nevertheless, it is recognised that the last edition of the ICOH code of ethics introduced some novelties: interdisciplinary approach to occupational medicine (psychology, ergonomics, environmental protection) and to continuous learning; proactive role of occupational physicians in improving workers’ safety and health; removal of language and cultural barriers and overcome of cultural differences; health surveillance based on scientific evidence and good practices; extended scope of application of the code of ethics to organisations and not only to OHPs.

Furthermore, the current main ethical principles of occupational health need continual updating and reformulation so as to keep abreast of developments in national legislations as well as changes in the workplace with the introduction of new ways of delivering work and new working processes that may lead to a depersonalization of the worker and his/her basic human needs [8,86].

There are some important limitations regarding our review. There is a possibility that we missed some articles not present in PubMed or Google Scholar; in addition, we did not quantitatively determine effect sizes across studies using a meta-analysis. Our intent was to provide a narrative review to underline the current state of literature.

Today’s globalized economy needs to take an integrated approach to assessing the importance of all three types of ethics: personal (individual), professional and institutional (Table 1).

The three types of ethics (personal, professional and institutional) may overlap in real life; professionals are influenced by their personal values and institutions are managed by individuals with their personal and professional values. In particular, many studies focused on the key role of “culture”, considered as an important carrier of determinants of morality of groups/tribes/regions/people/nations/socio-economic strata of populations, in the ethical decision making [31]. Several guidelines, however, emphasize the importance of keeping these three roles separate and independent.

## 5. Conclusions

Although the topic of ethical conflict in occupational health has been discussed since the 1970s and has received increased attention in recent years, there has been no systematic attempt to study the true extent of these problems and how they are resolved in practice [39]. In the process of management and adaptation to the changing world of work, ethics is often ignored even though there are several provisions in an organization’s code of manuals, ethics and ethical culture.

To this end, to deal with the complexity of today’s ever-shifting world of work with its new socio-demographic features and new technologies, the logic of an integrated approach must take account of the importance of all three types of ethics: personal (individual), professional and institutional. Before undertaking an analysis, it is usually necessary to map out the situational context and the norms involved. Discussion, analysis, problem solving, and decision making are critical to the ethical resolution of conflicts. It is important to define the problem, obtain a careful analysis of the facts (e.g., stakeholders involved or affected), check on the values involved in any possible decision or try to anticipate the implications of alternative decisions, evaluate cost-benefit of ethical decision [9,87].

Certain points of strength are useful and important for placing an ethical choice in its context. First of all, there is one’s own experience, and the knowledge and skills acquired from professional training and continued education. Another is the importance of working in a professional network, with a participatory, multidisciplinary outlook, so as to share all these features. Then there are the legal developments in each country, inspired by the general principles of safeguarding workers, and tackling the challenges posed by shifts in the world of work.

One must also bear in mind all the aspects that form barriers for correct professional ethics: these include conflicts of interest, failure to keep up to date, taking a “closed” attitude to one’s own single discipline, and discrimination. On this last point, social morals may foster discrimination against certain categories of workers, on the basis of their sex, nationality, religion, or political beliefs.

The most important “next step” useful to resolve ethical challenges should include:

(a) The development of a corpus of ethical principles that adequately consider the changing world of work, demographic shifts, new technologies and, more generally, the impact of globalization. Ethics code must be reviewed and updated providing indications for OHPs and serving as reference point against which to measure one’s own work;
(b) The introduction in the curricula of both medical undergraduates and postgraduates of ethic courses;
(c) A closer collaboration between OHPs and other key professionals. OHPs must seek the support and cooperation of employers, workers and their organizations, as well as of the competent authorities, professional and scientific associations and other relevant national and international organizations, for implementing the highest standards of ethics in occupational health practice [8];
(d) Develop scenarios that highlights ethical dilemmas which do not present easy policy choices. Scenarios are a useful instrument to provoke policymakers and other stakeholders, including industry, in considering the privacy, ethical, social and other implications of changing world of work, particularly new and emerging technologies [88].

## Figures and Tables

**Table 1:** Personal, Professional and Institutional ethics.

**(a). Target**			
	**Personal Ethics**	**Professional Ethics**	**Institutional Ethics**
**Person/body involved**	Individual	Expert	Institution, company, (and their boards and chief executive officers)
**Arena of operation**	Home, private life, community life	Workplace, association, public life	Public environment, business life, community
**(b). Philosophical and cultural bases, values, field of application**			
	**Personal ethics**	**Professional ethics**	**Institutional ethics**
**Philosophical and cultural basis**	Religious ethics, ethnicity, individual humanist ethics, or similar	Deontology	Deontology
**Field of application**	Family, close community, school, workplace	School, university, workplace, professional association, community	Institution, workplace, community, global economy
**Value content**	**Personal values**	**Professional values**	**Five principles of CSR & Global Compact:**
	Honesty	Fairness	Fair business
	Trustworthiness	Respect of autonomy	Accountability
	Respect	Beneficence	Transparency
	Responsibility	Non-maleficence	**Human rights (HR)**
	Integrity	Justice	Implementing HR
	Fairness	Competence	Acting against HR abuses
	Compassion, caring	Skill	**Fair employer**
	Courage	Confidentiality	Workers’ rights
			Elimination of inhuman labor
			Anti-discrimination
			**Environment**
			Precautionary principle
			Environmental responsibility and environment-friendly technologies
			**Anti-corruption**
**(c). Guidance and education**			
	**Personal ethics**	**Professional ethics**	**Institutional ethics**
**Learning arena**	Family, school, associations, church	Training institutions, schools, universities, polytechnics, professional stages	University, business school
**Guidance**	Guidance in general upbringing and school or religious education	Professional codes of conduct, Good practice guidelines, Helsinki Declaration, Council for International Organizations of Medical Sciences (CIOMS) guidelines	CSR, United Nations global compact

## Abbreviations

The following abbreviations are used in this manuscript: CIOMS: Council for International Organizations of Medical Sciences; CSR: Corporate Social Responsibility; DPR: Doctor-patient relationship; FOM—Faculty of Occupational Medicine; HR. Human rights; ICOH: International Commission on Occupational Health; ILO: International Labour Organization; OECD: Organisation for Economic Co-operation and Development; OHPs: Occupational health professionals; OSH: occupational safety and health; UART—Uniòn de Aseguradoras de Riesgos del Trabajo WCSDG—World Commission on the Social Dimension of Globalization.
